# A Superstable Homogeneous Lipiodol-Nanoformulation to Overcome the Dilemma of Interventional Embolization Chemotherapy

**DOI:** 10.3389/fbioe.2022.952194

**Published:** 2022-06-21

**Authors:** Yisheng Peng, Pan He, Xing Gao, Gang Liu, Hongwei Cheng

**Affiliations:** State Key Laboratory of Molecular Vaccinology and Molecular Diagnostics, Center for Molecular Imaging and Translational Medicine, School of Public Health, Xiamen University, Xiamen, China

**Keywords:** hepatocellular carcinoma, transcatheter arterial chemoembolization, drug delivery, drug dispersion, lipiodol

## Introduction

Hepatocellular carcinoma (HCC) is one of the leading cancer-related death, with morbidity and mortality rates increasing annually. As the most common treatment for HCC, transcatheter arterial embolization (TACE) plays an important role in HCC therapy (Singal et al., 2020). However, clinical practice has shown that the choice of embolic agents is limited by postoperative recanalization, non-targeted embolization, etc. (Chen et al., 2020b; Jiang et al., 2022). Furthermore, TACE also suffers from rapid drug release and insufficient therapeutic doses, which might contribute to drug resistance and poor therapeutic effects (Cao et al., 2021). Therefore, manufacture of the homogeneous drug dispersion in embolic agents is a revolutionary progress. Recently, He *et al.* reported a superstable homogeneous lipiodol-hydrophilic chemodrug formulation for TACE of HCC (He et al., 2022), which significantly enhanced the dispersion of chemotherapeutic drugs in lipiodol, thereby increasing the drug bioavailability of TACE in HCC patients and overcoming the shortcoming of rapid drug-releasee in clinic. Compared with the conventional surfactant emulsion strategy to increase drug dispersibility (Zhu et al., 2022), this superstable homogeneous intermixed formulation technology (SHIFT) is a green manufacture with any additions (Chen et al., 2020a; Cheng et al., 2020). And the chemotherapeutic drugs are assembled into nanostructures under the condition of cardon dioxide supercritical fluid. The specific nano-size effect offers the better dispersibility of chemotherapeutic drugs in lipiodol, suggesting this technology is universal in TACE. The chemotherapeutic drugs with nanostructures exhibit the better cellular uptake and sustained drug release properties. In general, this opinion prospects the interpretation of recent progress in the interventional embolization therapy of HCC, and focuses on the advantages of SHIFT technology in improving the stability of embolic reagents, improving the bioavailability of chemodrugs, and improving the prognosis of HCC.

## The Dilemma of TACE in Clinical HCC

Recent studies reported over 900,000 new HCC global cases by 2020, and by 2025, over one million people are expected to suffer with HCC every year (Siegel et al., 2022). For many years, surgical resection and liver transplantation have proved as primary methods for curative HCC treatment. However, liver decompensation after hepatectomy is a great concern for surgeons. Small-disseminated nidus or multi-center occurrences may exist before surgery, therefore the 5-year recurrence rate for HCC patients after surgery is close to 70%, and the 5-year survival rate is only 10% (Llovet et al., 2021). The long-term prognosis of HCC patients undergoing liver transplantation is superior to those patients with surgical resection. However, donor shortages often deprive HCC patients with optimal transplantation opportunities due to tumor progression. Therefore, main treatments for unresectable HCC patients are local ablation, chemotherapy, radiotherapy or interventional embolization, all of which are called palliative treatments. Among them, TACE is the most widely used in clinical practice.

In a normal liver, hepatic arterial blood supply accounts for approximately 25% and portal venous blood supply for approximately 75%, while in HCC tissue, hepatic artery blood supply accounts for approximately 90%, and portal vein blood supply is lower to 10% (Shiraishi et al., 2008). These blood flow parameters constitute the theoretical foundation of vascular interventional therapy. TACE exploits this by dissolving chemotherapeutic drugs, such as doxorubicin or carboplatin, in embolic agents and injecting them into tumor feeding arteries, causing serious tumor cytotoxicity, ischemia and hypoxia, while inhibiting and killing tumor cells (Wei and Zhang, 2021; Reig et al., 2022). Compared to systemic chemotherapy, TACE significantly increases drug concentrations in liver cancer tissue and reduces systemic adverse events. However, for unresectable HCC tumors with a diameter over 10 cm, these therapeutic effects remain largely unsatisfactory and may cause serious adverse reactions, including iodine oil ectopic embolism, oncolytic reactions, fever, liver abscess, bone marrow suppression after chemotherapy, and other risks (Kishore et al., 2020). One possible reason for this is that the current hand-emulsifier lipiodol with hydrophilic chemotherapy drugs such as doxorubicin, epirubicin, and fluorouracil, displaying the rapid drug release into systemic blood circulation after TACE (<30 min), which unfortunately elevate postoperative patient side effects and reduce therapeutic efficacy. Therefore, a novel technology is urgently required to stably disperse hydrophilic chemotherapeutic drugs in lipiodol to improve the therapeutic effects and safety of TACE treatments.

## Superstable Homogeneous Intermixed Formulation Technology for Enhanced TACE

**FIGURE 1:**
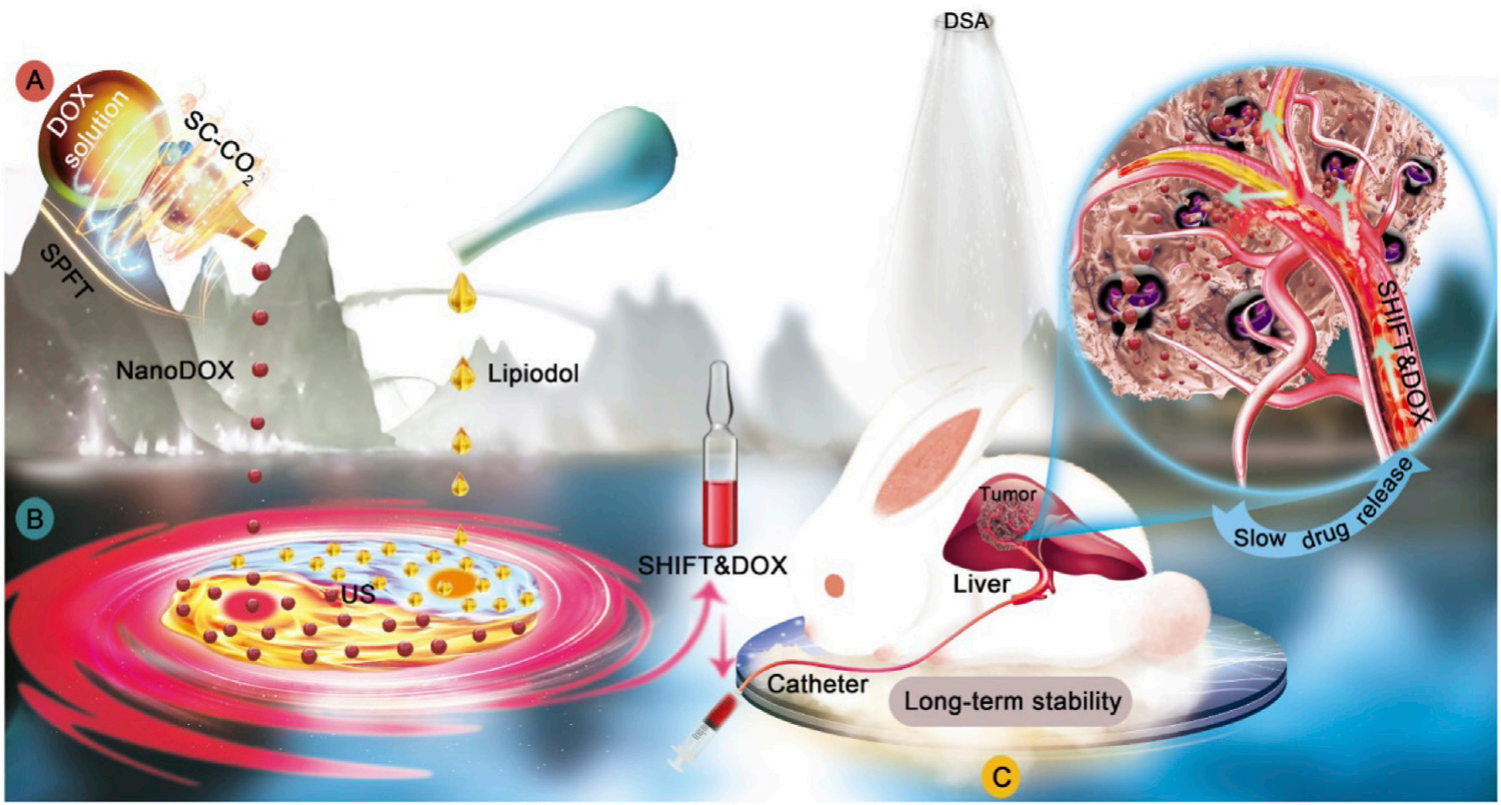
Illustration of superstable homogeneous lipiodol-chemodrug formulation in TACE. **(A)** SPFT was applied to produce nanoDOX with a nanoscale size and excellent uniformity. **(B)** nanoDOX was homogeneously dispersed into lipiodol *vi*a ultrasonication to achieve the superstable homogeneous lipiodol-DOX formulation (SHIFT&DOX). **(C)** The SHIFT&DOX embolic formulation showed the sustained drug release and enhanced therapeutic effects. Reproduced with permission (He et al., 2022). Copyright 2022, Ivyspring International Publisher.

SHIFT was developed to stably disperse chemotherapeutic drugs in lipiodol, overcoming the above mentioned issues during TACE (Figure 1). This technology skillfully combined lipiodol and hydrophilic chemotherapeutic drugs to prepare an effective and superstable homogeneous lipiodol and doxorubicin hydrochloride mixture (SHIFT and DOX), which was verified in several animal models, including decellularized liver, N1S1 rat orthotopic liver tumor and VX2 rabbit orthotopic liver tumor, showing improved clinical transformation and application value.

Doxorubicin (DOX), a hydrophilic chemotherapeutic drug, was transformed into pure nano-doxorubicin by supercritical technology and then dispersed in a stable and uniform manner in the lipiodol with a simple low-power ultrasound. In addition to improving the stability of doxorubicin in lipiodol, the SHIFT and DOX formulation did not contain any additional additives. These benefits have ensured its safety and effectiveness, with great prospects in translational medicine. When compared with traditional lipiodol chemotherapy, this strategy significantly reduced chemotherapy levels reaching the systemic circulation, while ultra-high sustained-release properties were maintained for up to 21 days, thereby increasing local drug concentrations and anti-tumor efficacy. Animal studies reported that SHIFT and DOX showed good drug-specific deposition and sustained drug-release effects, therapeutic effects, and safety in tumor areas. Therefore, SHIFT and DOX could address issues with drug burst release, poor stability, poor efficacy, poor repeatability, serious side effects in current conventional TACE. Additionally, for those patients who cannot undergo surgery, SHIFT and DOX could ameliorate the transformation efficiency of patients with large and advanced liver cancer, favoring HCC patients enduring curative tumor resection. Furthermore, for HCC patients with a high risk of tumor recurrence after surgery, retrospective studies have demonstrated that postoperative TACE treatment can reduce recurrence and prolong survival (Chen W. et al., 2020). Considering the better performance of SHIFT and DOX in nanoscale size, it is believed SHIFT and DOX could penetrate into tiny lesions and kill tumors, thereby reducing tumor recurrence and improving the prognosis of patients with large and advanced liver cancer.

## Discussion

With the application of nanotechnology in clinical cancer treatments, it has shown remarkable prospects in improving drug solubility, increasing drug bioavailability, enhancing therapeutic efficacy and reducing side effects. Nab paclitaxel is a good example of nanotechnology in cancer treatment, which combined paclitaxel with albumin protein to assemble into nanostructure, improving the water solubility of paclitaxel and avoiding the severe immune response caused by the addition of castor oil (Ma et al., 2021). Similar examples are irinotecan and mitoxantrone liposome complexes for the enhancement of tumor chemotherapy (Li et al., 2021). However, while utilizing nanotechnology to enhance the efficacy of drugs, the potential side effects of vector introduction are also worthy of vigilance. Therefore, pure drug nanomedicine has received more and more attention in recent years. On the one hand, it makes effective use of nanotechnology in drug delivery and efficacy enhancement, and on the other hand, it avoids potentials risks caused by the introduction of additional carriers. For the application, SHIFT technology is to exploit this mechanism of pure drug nano-assembly to prepare homogeneous lipiodol formulation.

In addition, SHIFT technology could also be used in other medical field fields. While not just used for mixing lipiodol and chemotherapeutics, it can be used for the superstable and uniform mixture of lipiodol and indocyanine green (ICG) to improve ICG fluorescence properties and guide the complete resection of liver cancer (Chen et al., 2020a; Zhang et al., 2021). Based on the SHIFT approach, it is believed that the superstable homogeneous lipiodol-hydrophilic drug can be applied to the interventional treatment of colorectal tumors and pancreatic tumor liver metastases, and even internal irradiation based on nuclides (Chen et al., 2022). In summary, the formulation of hydrophilic lipiodol chemotherapy drugs based on SHIFT technology has significant clinical perspectives and translational value for interventional embolization therapy. In future translational clinical applications, this technology would fill existing technology gaps and modify palliative treatment strategies for patients with advanced liver cancer.
